# The Additive Antinociceptive Effect of Resveratrol and Ketorolac in the Formalin Test in Mice

**DOI:** 10.3390/ph16081078

**Published:** 2023-07-28

**Authors:** Fidencio Abner Rojas-Aguilar, Alfredo Briones-Aranda, Osmar Antonio Jaramillo-Morales, Rodrigo Romero-Nava, Héctor Armando Esquinca-Avilés, Josué Vidal Espinosa-Juárez

**Affiliations:** 1 Sección de Estudios de Posgrado e Investigación, Escuela Superior de Medicina, Instituto Politécnico Nacional, Ciudad de Mexico 11340, Mexico; abnerrojasa06@gmail.com (F.A.R.-A.); roloromer@gmail.com (R.R.-N.); 2 Laboratorio de Farmacología, Facultad de Medicina Humana, Universidad Autónoma de Chiapas, Tuxtla Gutiérrez 29050, Chiapas, Mexico; alfred725@hotmail.com; 3 División de Ciencias de la Vida, Departamento de Enfermería y Obstetricia, Campus Irapuato-Salamanca, Universidad de Guanajuato, Irapuato 36500, Guanajuato, Mexico; oa.jaramillo@ugto.mx; 4 Escuela de Ciencias Químicas, Universidad Autónoma de Chiapas, Ocozocoautla de Espinosa 29140, Chiapas, Mexico; hesquinc@unach.mx

**Keywords:** resveratrol, ketorolac, isobologram, pain, gastric damage, drug interactions, mice

## Abstract

Pain represents one of the leading causes of suffering and disability worldwide. Currently available drugs cannot treat all types of pain and may have adverse effects. Hence, the use of pharmacological combinations is an alternative treatment strategy. Therefore, this study aimed to evaluate the combination of resveratrol and ketorolac through isobolographic analysis. CD1 mice were used to study the antinociceptive effect of this combination using the formalin test and the study was divided into two phases. In the first phase, four individual doses of each drug were evaluated, totaling eight testing groups. From these data, the median effective doses (ED_50_) of each drug were calculated. In the second phase, four testing groups were used to evaluate the combination of sub-doses of both drugs and obtain the experimental ED_50_. To evaluate gastric damage, five groups were employed, including indomethacin, vehicle, resveratrol, ketorolac, and combined resveratrol and ketorolac groups. Stomach samples from the mice were taken after 5 h of treatment, and the area of the ulcers was determined. Resveratrol plus ketorolac elicited a reduction in nociceptive behavior during both phases of the formalin test, and isobologram analysis revealed that the theoretical and experimental ED_50_ values of resveratrol and ketorolac did not differ significantly, implying an additive interaction between the drugs. Additionally, the drug combination did not generate gastric ulcers, thus enhancing the desired effects without increasing the adverse effects. Consequently, these findings substantiate the efficacy of the resveratrol and ketorolac combination in the formalin test, thereby highlighting its potential as a viable alternative for alleviating pain.

## 1. Introduction

Pain constitutes a profoundly incapacitating condition in many cases, exerting a detrimental influence on the quality of life of those affected. Consequently, it is one of the foremost reasons individuals pursue medical care [1,2].

The International Association for the Study of Pain (IASP) defines pain as an unpleasant sensory and emotional experience associated with, or resembling that associated with, actual or potential tissue damage [3]. However, there are various types of pain, including nociceptive, inflammatory, and neuropathic pain [4]; this poses a challenge to proper treatment when multiple pain types coexist, thereby leading to polypharmacy and the escalation of medication doses; this results in an increased risk of adverse events [5]. 

The pharmacological treatment of pain depends on different circumstances, such as chronicity and intensity. Generally, for mild to moderate acute or chronic pain, paracetamol, nonsteroidal anti-inflammatory drugs (NSAIDs), or short-acting opioids are used. For intense pain, more potent drugs, such as opioids, are employed. [6]. Nonetheless, NSAIDs, which are often the first choice for treatment, can have adverse effects, such as [7] renal [8], or cardiovascular alterations [9]. This poses a challenge when relying on a single analgesic agent that targets a specific pain pathway to achieve effective pain relief. Higher doses may be required to achieve the desired pain reduction, which, in turn, increases the risk of side effects [10].

Given these considerations, the use of pharmacological combinations is necessary for pain management. The goal of using medications that act on multiple signaling pathways is to maximize therapeutic coverage while minimizing side effects [10]. In this study, ketorolac, an NSAID, was used. Ketorolac is known for its potent analgesic activity and moderate anti-inflammatory action [11]; similar to other drugs in its class, its mechanism of action involves cyclooxygenase (COX) inhibition, thereby preventing the production of inflammatory mediators, such as prostaglandins [12]. As a result, ketorolac is indicated for the treatment of moderately intense acute pain [13].

Resveratrol is an alternative molecule for treating pain; it is a natural polyphenol first characterized in 1939 from the roots of *Veratrum grandiflorum* (Maxim ex. Baker) Loes [14]. Resveratrol has been called the “elixir of life” due to its antioxidant [15,16] anti-inflammatory [17] and analgesic [18] properties, as indicated by reports in both animals [19,20] and humans [21]. Additionally, resveratrol acts on various pathways related to pain perception and transmission, such as suppressing inflammatory mediator production through inhibiting NF-κB translocation to the nucleus [22], as well as inhibiting COX enzymes [23]. Additionally, it has a potent inhibitory effect on transient receptor potential cation channel subfamily A member 1 (TRPA1) and transient receptor potential cation channel subfamily V member 1 (TRPV1), channels involved in nociceptive signaling [24]. Furthermore, resveratrol had a favorable safety profile in many studies, making it a promising candidate for the development of safer and more effective analgesic therapies [15].

The combination of resveratrol and ketorolac as an analgesic represents a novel and promising approach in the search for more effective therapeutic strategies for pain relief. Both compounds have analgesic properties individually, but when combined, their synergistic potential could provide additional benefits, such as increased efficacy and reduced unwanted side effects. This innovative approach opens new possibilities for the development of safer and more efficient analgesic therapies, which could lead to a significant improvement in the quality of life of patients with chronic or acute pain. Therefore, the objective of the present research was to evaluate the effect of the combination of ketorolac with resveratrol using an isobolographic analysis in the formalin test in mice. Before that, it was determined the drug association’s feasibility by an in silico study.

## 2. Results

### 2.1. In Silico Analysis

#### 2.1.1. Molecular Targets

**Figure 1:**
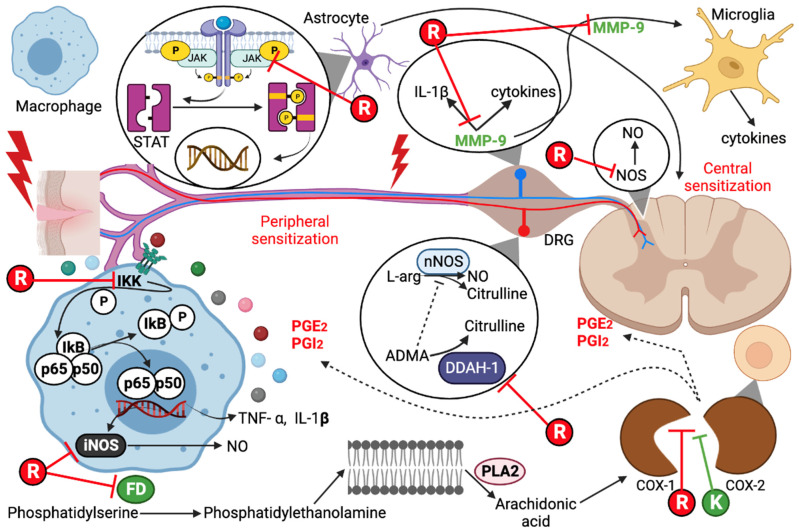
Pathways of pain inhibition by resveratrol (R) and ketorolac (K). COX, cyclooxygenase; iNOS, inducible nitric oxide synthase; MMP-9, matrix metallopeptidase 9; ADMA, asymmetric dimethylarginine; DDAH-1, dimethylargininase; PLA_2_, phospholipase A_2_; PGE_2_, prostaglandin E_2_; PGI_2_, prostacyclin; FD, phosphatidylserine decarboxylase; DRG, dorsal root ganglion; nNOS, neuronal nitric oxide synthase; JAK, Janus kinase; NO, nitric oxide.

The possible effectiveness of the combination of ketorolac and resveratrol for pain treatment was evaluated through an in silico analysis using PASS online and SwissTargetPrediction. The results show that resveratrol can target both isoforms of the COX enzyme (>99%). Additionally, several molecular targets with high probabilities (>50%) were identified in Table 1, indicating that the antioxidant resveratrol could inhibit pain through these targets. On the other hand, ketorolac is an inhibitor of both COX isoforms (>99%) (Table 2). All the molecular targets presented in Table 1 and Table 2 involve pain signaling. Based on these tables, Figure 1 visually shows how both ligands could interact to inhibit pain. These approximations give us an idea of the potential these drugs could have if they are combined, expanding the range of pharmacological targets to counteract pain.

#### 2.1.2. In Silico Analysis of CYP-Dependent Metabolism

The predictions regarding resveratrol and ketorolac are divided into substrates or inhibitors of CYP enzymes. Table 3 displays the probabilities of the drugs acting as CYP isoform inhibitors, while Table 4 shows the results for the drugs as enzyme substrates. Both analyses were conducted using the admetSAR web server.

The results indicate that resveratrol exhibits a high probability (>0.70) of inhibiting four CYP isoforms (1A2, 3A4, 2C19 and 2C9). Conversely, ketorolac was not identified as a substrate for these isoforms. Ketorolac demonstrates a probability of 0.5483 of inhibiting CYP1A2. However, the programs did not provide evidence regarding whether resveratrol acts as a substrate for CYP1A2. Notably, both drugs exhibited inhibitory effects on CYP1A2; ketorolac showed a relatively lower probability (<0.5483).

### 2.2. Evaluation of the Antinociceptive Activity of Individual Drugs in the Formalin Test

**Figure 2:**
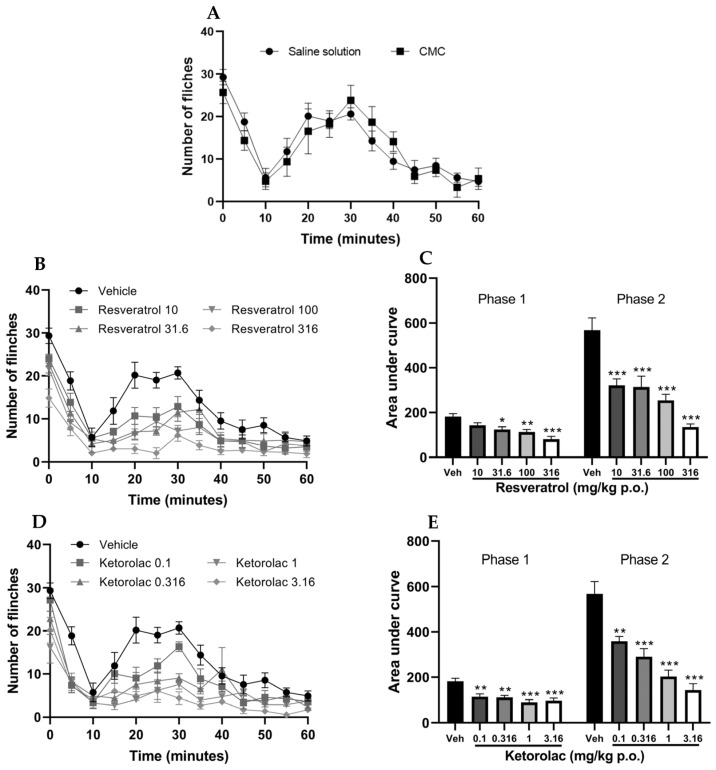
Temporal course of the number of limb flinches induced by formalin administration after the administration of vehicles (saline solution 0.9% and carboxymethylcellulose 0.1%) (**A**), resveratrol (10, 31.6, 100, and 316 mg/kg) (**B**) and ketorolac (0.1, 0.316, 1, and 3.16 mg/kg) (**D**). The bar graph shows the area under the curve of (**C**) resveratrol and (**E**) ketorolac for the two phases in formalin test. The mean value plus standard error of the mean (S.E.M.) is presented for six animals per group, with each dose representing a separate group. * *p* < 0.05, ** *p* < 0.01, *** *p* < 0.001 compared to the vehicle, based on one-way analysis of variance (ANOVA), followed by Tukey’s post hoc test.

The subcutaneous administration of 2% formalin in the hind limb of mice treated with the vehicle resulted in a nociceptive biphasic response that is characteristic of the test. It should be noted that the administration of both saline solution and carboxymethylcellulose yielded similar results (Figure 2A). As no significant differences were observed in the effects of both vehicles, it was decided to use only one of them (saline solution) for the graphical representations.

The temporal course of the four doses of resveratrol and the vehicle is depicted in Figure 2B. The number of limb flinches in mice decreased during both phases. The area under the curve (AUC) was calculated for both phases of the test; the neurogenic phase (0–10 min) and the inflammatory phase (15–60 min). Figure 2C illustrates the area under the curve (AUC) for each dose, with a lower AUC indicating a reduction in nociceptive behavior. The graph also demonstrates that the AUC of the four doses is significantly different from the vehicle in both phases (*p* < 0.05), except for the 10 mg/kg dose in the first phase, which was not significantly different from the vehicle. The percentages of antinociception were 22.25, 31.65, 37.61, and 55.27% for the first phase, and 43.47, 44.72, 55.27%, and 76.17% for the second phase, respectively.

The temporal course of the four evaluated doses of ketorolac is shown in Figure 2D, illustrating a reduction in the number of limb flinches in mice during both phases compared to the vehicle. Subsequently, the AUC was determined (Figure 2E), revealing that the AUCs of the four different doses were statistically distinct from the vehicle group (*p* < 0.01), indicating the antinociceptive effect caused by ketorolac in both phases. The percentages of antinociception induced by the doses (0.1, 0.316, 1, and 3.16 mg/kg) assessed in the first phase were 22.25, 37.84, 39.44, 50.91, and 47.01%, respectively. In the second phase, the corresponding percentages were 36.87, 48.97, 64.22, and 74.78%, respectively.

Based on the dose–response curve constructed using the percentage of antinociception considering the maximum possible effect of both phases of the test, the maximum effect of resveratrol was 71.12% with a dose of 316 mg/kg. In comparison, ketorolac exhibited an efficacy of 66.6% with a dose of 3.16 mg/kg. Consequently, the median effective dose (ED_50_) values for resveratrol and ketorolac were 59.9 ± 12.36 and 0.44 ± 0.04 mg/kg, respectively. From these data, four subdoses were analyzed in combination, as shown in Table 5.

### 2.3. Evaluation of the Antinociceptive Activity of Drug Combinations in the Formalin Test

**Figure 3:**
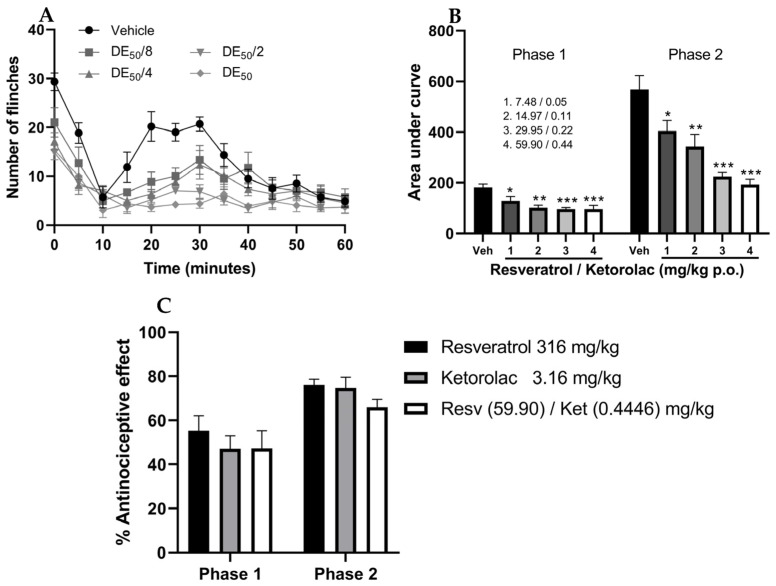
Temporal course of the number of limb flinches in the formalin test after the administration of (**A**) the resveratrol–ketorolac combination (7.54, 15.08, 30.17, and 60.34 mg/kg) and the vehicle. (**B**) The bar graph shows the area under the curve of each combination. (**C**) Antinociceptive effect of the highest doses of the drugs used individually and the highest doses used in combination. Mean ± S.E.M. of 6 animals per group is presented in all cases, with each dose corresponding to a group. * *p* < 0.05, ** *p* < 0.01, *** *p* < 0.001 compared to the vehicle, according to one-way analysis of variance (ANOVA), followed by Tukey’s post hoc test.

As observed in Figure 3A, the temporal course of the four doses in combination resulted in a decrease in the number of limb flinches in mice compared to the vehicle. Furthermore, Figure 3B represents the AUC of all combinations (7.54, 15.08, 30.17, and 60.34 mg/kg), indicating that all four combinations were significantly different from the vehicle group (*p* < 0.05). The percentages of antinociception in the first phase were 29.35, 44.26, 47.24, and 47.24%, while in the second phase, they were 28.88, 39.66, 60.48, and 65.98%, respectively.

In Figure 3C, the effects of the highest doses administered individually (resveratrol, 316 mg/kg; ketorolac, 3.16 mg/kg) are compared with the ED_50_ doses of their combination (60.3446 mg/kg). The percentages of antinociception in the neurogenic phase were 55.27, 47.01, and 47.24%, respectively, while in the second phase, they were 76.17, 74.78, and 65.98%, respectively. No statistically significant differences (*p* < 0.05) were observed in the antinociceptive effects between groups; this demonstrates that to achieve effects similar to the highest individual doses, approximately five times less resveratrol and seven times less ketorolac is necessary when combined.

### 2.4. Isobolographic Analysis of the Combination of Resveratrol with Ketorolac

**Figure 4:**
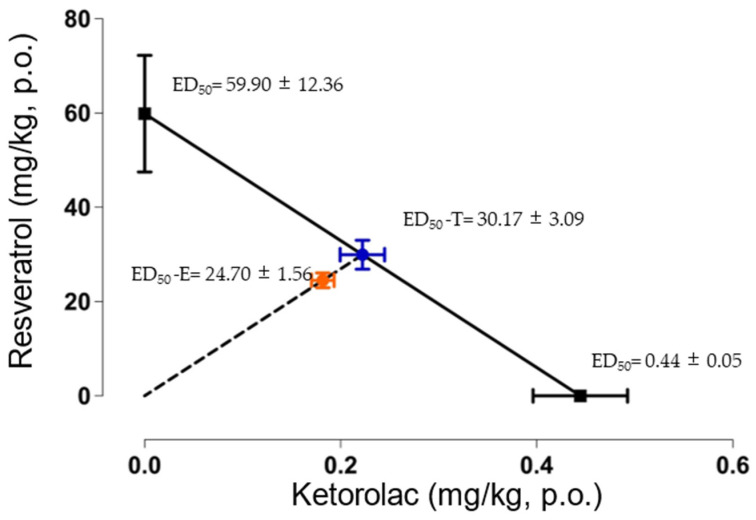
Isobologram of the antinociceptive interaction between resveratrol and ketorolac in a dose ratio of 1:1. The points on the y-axis represent the ED_50_ of resveratrol, while the points on the x-axis represent the ED_50_ of ketorolac. The diagonal line connecting the ED_50_ values of resveratrol and ketorolac represents the line of additivity. The midpoint (blue point) between the two drugs corresponds to the theoretical ED_50_ (ED_50_-T), and the point below represents the experimental ED_50_ (ED_50_-E). The horizontal and vertical bars indicate the standard error of the mean (S.E.M). The ED_50_-T and ED_50_-E were not statistically significantly different according to a Student *t*-test, indicating an additive interaction.

After determining the ED_50_ values for individual drug administration, the theoretical ED_50_ (ED_50_-T) was obtained (30.17 ± 3.09 mg/kg). The experimental ED_50_ (ED_50_-E) was 24.70 ± 1.56 mg/kg. Figure 4 presents the isobologram, demonstrating the statistical comparison between the ED_50_-T and ED_50_-E, revealing no statistically significant differences between them. Furthermore, an interaction index of 0.81 was calculated, indicating an additive interaction between resveratrol and ketorolac.

### 2.5. Evaluation of Gastric Damage Caused by the Drugs

**Figure 5:**
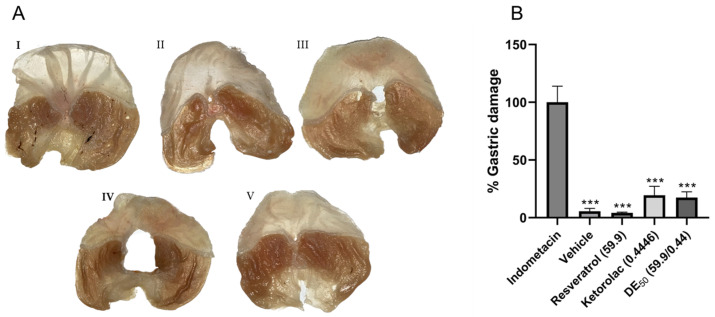
(**A**) The appearance of the stomachs after 5 h of treatment. (I) indomethacin (25 mg/kg), (II) vehicle, (III) resveratrol (59.9 mg/kg), (IV) ketorolac (0.44 mg/kg), and (V) resveratrol–ketorolac combination (59.9 + 0.44 mg/kg). (**B**) Percentage of gastric damage of treatments compared to the group used as the reference for 100% damage (indomethacin). Gastric lesions were quantified using the ImageJ program. The bars represent each group’s mean ± SEM of gastric lesions (n = 6). *** *p* < 0.001 according to one-way ANOVA followed by Tukey’s post hoc test.

In order to identify possible adverse gastrointestinal effects caused by the individual drugs and their combination, the area of gastric ulcers was quantified. Within 5 h of treatment in the different groups that received resveratrol, ketorolac, the combination, and the vehicle, the minimum gastric damage was significantly different (*p* < 0.001) compared to the reference group with 100% gastric damage (indomethacin-treated group). Images of gastric lesions are shown in Figure 5, where the stomach damage was caused by indomethacin (Figure 5A, panel I), in contrast to stomachs treated with the vehicle and resveratrol (Figure 5A, panels II and III, respectively), where gastric damage was minimal. In Figure 5B, the differences in the percentage of gastric damage in each group compared to the reference group are represented. The percentages of gastric lesions were 5.53 ± 2.5, 4.17 ± 0.66, 19.45 ± 7.71, and 17.46 ± 4.96% for the groups that received the vehicle, resveratrol, ketorolac, and the combination, respectively. The percentage of lesions in the ketorolac and combination groups represents approximately one-fifth of the damage observed in the indomethacin group. No statistically significant differences were found between the groups administered the vehicle, resveratrol, ketorolac, and the combination.

## 3. Discussion

Resveratrol has been the subject of research due to its various therapeutic properties [16,17,18]; however, the combined effects of resveratrol and ketorolac on pain management have not been thoroughly examined. Therefore, this analysis was crucial to understand and provide insights into the pathways through which these drugs can exert an antinociceptive effect.

In silico analysis of the pharmacodynamics of these drugs revealed their potential antinociceptive effects, indicating novel targets for resveratrol. There is substantial evidence in the literature for the inhibitory effect of resveratrol on the majority of these targets [23,25,26,27,28,29,30], which explains the antinociceptive action of the drug. However, there is no evidence to suggest that resveratrol acts as an inhibitor of phosphatidyl-serine decarboxylase (PISD) or dimethylargininase (DDHA). PISD plays a role in phosphatidylserine decarboxylation to produce phosphatidylethanolamine, which is a key phospholipid in cell membranes [31,32]. Upon stimulation, the enzyme phospholipase A2 triggers the release of arachidonic acid from membrane phospholipids [33], suggesting that by inhibiting the PISD enzyme, no phosphatidylethanolamine (PE) is produced, thereby preventing arachidonic acid production and inhibiting the formation of inflammation-mediating molecules that contribute to pain generation.

DDAH is an enzyme that utilizes asymmetric dimethylarginine (ADMA) as its substrate. ADMA, which bears structural similarity to L-arginine, competes for the active site of nitric oxide synthase (NOS), thereby inhibiting nitric oxide production. [34,35,36]. According to the prediction results, resveratrol could be an DDAH inhibitor, and by inhibiting this enzyme, the ADMA concentration would be increased, thus blocking NOS activity [35,37]. This suggests that resveratrol could indirectly inhibit Nitric Oxide (NO) production, thereby preventing a nociceptive signaling pathway mediated by NO [38]. In contrast to the above, resveratrol can restore DDHA activity in bovine endothelial cells [39]. It is important to note that ADMA inhibits both neuronal (nNOS) and endothelial NOS (eNOS) [40,41], which suggests that resveratrol may not be entirely innocuous. Despite these reports, ADMA also exhibits a higher affinity for inhibiting nNOS than eNOS [40,41,42]. Moreover, neuronal ADMA upregulation dramatically suppressed NO-mediated excitotoxic injury, offering a novel therapeutic approach [34]. Additionally, nNOS activation and subsequent NO production can lead to spinal hyperexcitability and heightened pain sensation [35]. Therefore, it is important to confirm whether resveratrol inhibits DDAH and PISD, as these pathways could represent novel mechanisms by which resveratrol may act to inhibit pain.

A high probability (>99%) of ketorolac being an inhibitor of cyclooxygenases was obtained, which is widely supported by various studies [11,12,43]. Considering the in silico results of the molecular targets of both drugs, it is evident that resveratrol exerts pleiotropic effects by inhibiting various molecular targets. Additionally, both ketorolac and resveratrol demonstrate analgesic activities, indicating the potential for a pharmacodynamic interaction when administered together.

The in silico predictions of CYP-dependent metabolism indicated that resveratrol does not undergo phase 1 metabolism as a substrate for any isoform. This is consistent with previous studies. The resveratrol metabolites reported in the literature, such as glucuronides, are formed through the activity of the enzymes UGT1A1, UGT1A9, UGT1A6, UGT1A7, and UGT1A10 [44,45]. Additionally, resveratrol acts as a substrate for the enzymes SULT1A1, SULT1A2, and SULT1A3, leading to resveratrol sulfate formation [46,47]; all of these enzymes are involved in phase 2 metabolism.

Regarding the results of resveratrol as an inhibitor of CYP2C9, 3A4, and 2C19, the data are consistent with previous in vitro [48] and in vivo [49] studies; however, despite the predictions indicating that resveratrol does not inhibit CYP2D6, both studies mention that resveratrol inhibits this isoform [48,49]. 

Analysis revealed that ketorolac is neither a substrate nor an inhibitor of any of the five CYP enzymes. Therefore, the in silico analysis found no pharmacokinetic interaction regarding metabolism between ketorolac and resveratrol. However, it was reported that ketorolac is a substrate for CYP2C8, CYP2C9, and UGT2B7 enzymes [50]. Furthermore, in a study involving ketorolac administration to healthy volunteers, *p*-hydroxy-ketorolac was identified as a metabolite [51,52]; it is reasonable to assume that ketorolac is indeed a substrate for phase 1 enzymes (CYP2C8 and CYP2C9). Furthermore, as previously observed, resveratrol inhibits CYP2C9 [48,49], which indicates a positive pharmacokinetic interaction between both drugs, suggesting that when administered in combination, a lower dose of ketorolac may be sufficient to achieve therapeutic effects. 

The efficacy of resveratrol in pain relief has been reported in previous studies [19,20,53,54], which is consistent with the findings of the present investigation. Nonetheless, some studies [53,55] showed antinociceptive effects of resveratrol only in the second phase, in contrast to the current findings where a biphasic reduction in nociceptive behavior was observed. The differences in our research are likely attributable to methodological differences such as the route of administration (local vs. systemic). Additionally, previous reports suggest that resveratrol exerts antinociceptive effects by inhibiting TRPA1 [56] and TRPV1 [24] channels, both of which are involved in pain generation during the first phase [57,58,59] of the formalin test. Thus, the inhibition of these receptors by resveratrol may explain the antinociceptive effect observed in the first phase of our investigation.

In this study, we corroborate that ketorolac significantly reduces nociceptive behavior in the formalin test, which is consistent with studies [60,61,62,63] which reported effects in both phases of the test. Nociceptive behavior in the first phase of the formalin test can be attenuated by centrally acting drugs [64,65,66]. Previous research has demonstrated that ketorolac exerts analgesic effects at the central level [67,68,69], possibly through the involvement of descending modulatory systems [69]. This explains the analgesic effect observed in the present study by the action of ketorolac in the first phase of the formalin test.

In the additive interaction observed in the combination of resveratrol and ketorolac, the maximum antinociceptive effect was achieved with the higher doses of resveratrol and ketorolac, as well as the combination. Despite the smaller doses in the combination, no significant differences were found in the antinociceptive effect. The synergistic interaction index also demonstrated an additive interaction between ketorolac and resveratrol. Both drugs have been previously evaluated in combination with other medications. For example, resveratrol exhibited synergistic interactions with diclofenac, benfotiamine [20], and morphine [70] in acetic acid-induced constriction and hot plate models, respectively. As for ketorolac, previous research indicates synergistic interactions with methyl eugenol [63] and essential oils from *Syzygium aromaticum* and *Rosmarinus officinalis* [71] in the formalin test, as well as a synergistic interaction with tramadol in the pain induced functional impairment model in rats [72].

One of the main adverse effects of NSAIDs is gastric ulcers, associated with their effects as COX-1 inhibitors [73]. Despite increasing the antinociceptive effects in combination, our results show that gastric lesions were not exacerbated. It has been reported that resveratrol acts as a protective and therapeutic agent against oxidative gastric damage, which could contribute to the gastric effects observed with the administration of the combination [74,75]. 

## 4. Materials and Methods

### 4.1. In Silico Analysis

The SMILES (Simplified Molecular Input Line Entry System) codes [76] for each drug was obtained from PubChem; these codes are essential for conducting in silico analysis. The tests were divided into two phases. In the first part, the online programs PASS (Way2Drug) [77] and SwissTargetPrediction [78] were used to obtain the probabilities of interaction with specific molecular targets and identify potential signaling mechanisms for pain inhibition. The admetSAR [79] server was used for the second part of the analysis (accessed on 10 November 2022). To determine the probabilities of the drugs as substrates or inhibitors based on five CYP isoforms (3A4, 1A2, 2C9, 2C19, 2D6) to detect potential pharmacokinetic interactions at the metabolism level. The results are expressed as probabilities (P) on a scale from 0 to 1, where 1 indicates that the event is very likely to occur and 0 indicates that it is very improbable. Only predictions with a probability of occurrence >50% (>0.5) were considered.

### 4.2. Animals

For the experimental tests, 108 male CD1 mice weighing 25–30 g were used. The rodents were kept under controlled humidity conditions and a 12 h light/dark cycle, with ad libitum access to water and food, except for food withdrawal hours before the experiment (12 h for the formalin test; 18 h for gastric damage determination). The use and handling of the animals were carried out following the ethical guidelines for experimental pain research in animals proposed by the International Association for the Study of Pain [80] and under regulations established in the Official Mexican Standard for the care and use of laboratory animals (NOM-062-ZOO-1999). The experimental procedures were approved by the research committee of the Autonomous University of Chiapas (Approval date, 7 November 2022; protocol number, 03/ECQ/RPR/066/22). The number of animals used was kept to a minimum. Each animal was utilized only once for the experimentation. For the mice evaluated in the formalin test, they were euthanized by cervical dislocation.

### 4.3. Drugs

Ketorolac (PHARMAlife, Zapopan, Jalisco, Mexico, CAS 74103-06-3) and resveratrol (PlantPills, Nottingham, UK, CAS 501-36-0) were used to evaluate antinociception, while indomethacin (BIORESEARCH, Naucalpan de Juárez, Edo. de México, Mexico, CAS 53-86-1) was used as an inducer of gastric damage. Ketorolac and indomethacin were dissolved in an isotonic saline solution (0.9% NaCl, PiSA Laboratories, Mexico City, Mexico). Due to the insolubility of resveratrol in saline solution, it was suspended in 0.1% carboxymethylcellulose (Sigma-Aldrich, St. Louis, MO, USA, CAS 9004-32-4). The substances were administered orally (p.o.) in a volume of 1 mL/100 g of body weight. All preparations were made minutes before administration.

### 4.4. Formalin Test

**Figure 6:**
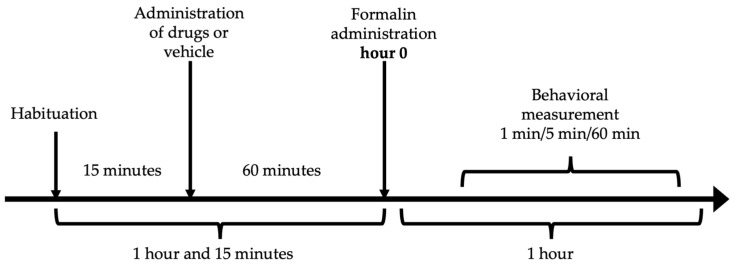
Experimental time sequence of the formalin test.

The antinociceptive effect was measured by the formalin test [81]. Each mouse was placed inside an acrylic cylinder (30 × 30 × 40 cm) with mirrors mounted on the back for 15 min to allow habituation. After this exploration of the novel environmental concluded and the rodent appeared comfortable and not stressed. Subsequently, the administration of resveratrol (10, 31.6, 100 and 316 mg/kg), ketorolac (0.1, 0.316, 1 and 3.16 mg/kg), the vehicle, or the combination (7.54, 15.1, 30.17 y 60.34 mg/kg) was performed, considering a latency period of 60 min. The individual doses of resveratrol and ketorolac were taken according to previously conducted research [20,72]. After the designated time, 20 μL of 2% formalin was administered to the dorsal surface of the mouse’s right hind limb and the mouse was placed back inside the cylinder. Only the number of paw flinches was counted as a measure of nociception during 1 min, every 5 min for 60 min (Figure 6). The response to the stimulus was analyzed in two phases: the first phase ranging from 1 to 10 min (neurogenic phase) and the second phase from 15 to 60 min (inflammatory phase). All evaluations were performed in real-time by a single researcher in a blinded manner. Throughout the assessment, the mice remained inside the observation chamber.

The time course curve for each drug dose was constructed by plotting the number of limb shakes induced by formalin as a function of time. The area under the curve (AUC) for the formalin phases was calculated using the trapezoidal method [82]. The percentage of the antinociceptive effect was calculated from the AUC using the following equation:

$$\%\;\mathrm{Antinociception}=\left(\frac{{\mathrm{AUC}}_{\left(\mathrm{control}\;\mathrm{group}\right)}-{\mathrm{AUC}}_{\left(\mathrm{test}\;\mathrm{drug}\right)}}{{\mathrm{AUC}}_{\left(\mathrm{control}\;\mathrm{group}\right)}}\times 100\right)$$

### 4.5. Isobolographic Analysis

Isobolographic analysis was performed using the ED_50_ values of the drugs administered individually and in combination, calculated from the dose–response curves. The isobologram was constructed by plotting the ED_50_ of ketorolac on the x-axis and the ED_50_ of resveratrol on the y-axis to obtain the theoretical additive line. The midpoint between the two doses represents the ED_50_-T [83]. From the individual ED_50_ values of each drug, four sub-doses were obtained and evaluated in the formalin test to obtain the dose–response curve of the combination and calculate the ED_50_-E. This ED_50_-T was compared with the ED_50_-E using a Student *t*-test. The interaction index was measured as described by Tallarida. [84], where values close to 1 indicate an additive interaction, values >1 correspond to an antagonistic interaction (subadditive), and values <1 indicate a synergistic interaction (superadditive).

### 4.6. Gastric Damage Assessment

The animals had an 18 h fasting period, followed by oral administration of the ED_50_ of resveratrol (59.9 mg/kg), ketorolac (0.4446 mg/kg), resveratrol–ketorolac (59.9 + 0.4446 mg/kg, respectively), indomethacin (25 mg/kg), and vehicle. A second administration was performed at the same doses 2.5 h later. Later, the mice were euthanized, and their stomachs were removed. The stomachs were rinsed with saline solution, and the interior was filled with 2% formaldehyde [85] followed by immersion in formaldehyde in the same proportion for 30 min. Subsequently, the stomachs were opened along the greater curvature for better extension and washed with saline solution to remove gastric content. The stomachs were photographed using a digital camera, and the images were analyzed with the ImageJ software version 1.53t (Wayne Rasband, National Institute of Health, Bethesda, MD, USA). The area of gastric damage was measured in millimeters [86]. The following equation was used to calculate the total percentage of gastric lesions [87]:

$$\%\;\mathrm{Gastric}\;\mathrm{damage}=100-\left(\frac{{\mathrm{Ulcer}\;\mathrm{area}}_{\left(\mathrm{indomethacin}\right)}-{\mathrm{AUC}}_{\left(\mathrm{test}\;\mathrm{drug}\right)}}{{\mathrm{AUC}}_{\left(\mathrm{indomethacin}\right)}}\times 100\right)$$

The administered doses of ketorolac, resveratrol, and ketorolac–resveratrol were the ED_50_ values previously calculated. The administration of indomethacin (25 mg/kg) was used as a reference for 100% gastric damage [85].

### 4.7. Statistical Analysis

The data from the evaluations of the antinociceptive effect in the formalin and the gastric damage are expressed as the mean ± standard error of the mean (SEM) of six animals per group. First, a normality test using the Shapiro–Wilk method was conducted on the data to ensure its adherence to a normal distribution. Subsequently, a one-way analysis of variance (ANOVA) was performed to determine any statistical differences, followed by a Tukey post hoc test for making multiple group comparisons. In the context of comparing the theoretical ED_50_ to the experimental ED_50_ from the isobologram, a Student *t*-test was employed. For statistical analysis, *p* < 0.05 was considered statistically significant. The analyses were performed using GraphPad Prism 6.0 software (SPSS Inc., Chicago, IL, USA).

## 5. Conclusions

This research demonstrates the antinociceptive efficacy of both resveratrol and ketorolac in the formalin test, highlighting their potential as analgesic agents with multi-pathway actions. The additive interaction observed with the resveratrol–ketorolac combination further suggests its promising role as an alternative for pain treatment and relief. These findings present a novel path for the development of more effective and safer analgesic therapies. The combination of resveratrol and ketorolac shows great promise as a potential clinical intervention, opening up new possibilities for future investigations and applications in pain-management strategies.

## Figures and Tables

**Table 1:** Molecular targets of resveratrol and their probabilities.

Probability	Molecular Target	Common Nomenclature	Software
1.0	Cyclooxygenase 1	COX-1/PTGS1	SwissTargetPrediction
1.0	Cyclooxygenase 2	COX-2/PTGS2	SwissTargetPrediction
0.912	Janus kinase 2 expression inhibitor	JAK2	PASS Online
0.773	Phosphatidylserine decarboxylase inhibitor	PISD	PASS Online
0.772	Matrix metallopeptidase 9 expression inhibitor	MMP9	PASS Online
0.681	Dimethylargininase inhibitor	DDAH	PASS Online
0.654	Tumor necrosis factor expression inhibitor	TNF	PASS Online
0.603	Nitric oxide synthase 2 expression inhibitor	NOS2	PASS Online
0.587	Transcription factor RelA expression inhibitor	RelA/p65	PASS Online

**Table 2:** Molecular targets of ketorolac and their probabilities.

Probability	Molecular Target	Common Name
1.0	Cyclooxygenase 1	COX 1/PTGS1
1.0	Cyclooxygenase 2	COX 2/PTGS2

**Table 3:** Probability of inhibition of resveratrol and ketorolac on CYP isoforms.

Inhibitor			
CYP Isoform	admetSAR	% Probability	Drugs
CYP2D6	No	0.9226	Resveratrol
	No	0.9333	Ketorolac
CYP3A4	Yes	0.7539	Resveratrol
	No	0.9621	Ketorolac
CYP1A2	Yes	0.9106	Resveratrol
	Yes	0.5483	Ketorolac
CYP2C19	Yes	0.8052	Resveratrol
	No	0.9225	Ketorolac
CYP2C9	Yes	0.7068	Resveratrol
	No	0.9081	Ketorolac

**Table 4:** Analysis of resveratrol and ketorolac as substrates of CYP isoforms.

Substrate			
CYP Isoform	admetSAR	% Probability	Drugs
CYP2D6	No	0.6927	Resveratrol
	No	0.8801	Ketorolac
CYP3A4	No	0.7342	Resveratrol
	No	0.6185	Ketorolac
CYP1A2	--	--	Resveratrol
	--	--	Ketorolac
CYP2C19	--	--	Resveratrol
	--	--	Ketorolac
CYP2C9	No	0.5955	Resveratrol
	No	0.6206	Ketorolac

**Table 5:** Doses of resveratrol and ketorolac evaluated in combination at a 1:1 ratio.

Combination	Dose		
	Resveratrol mg/kg	Ketorolac mg/kg	Total mg/kg
ED_50_ + ED_50_	59.9	0.44	60.34
(ED_50_ + ED_50_)/2	29.95	0.22	30.17
(ED_50_ + ED_50_)/4	14.97	0.11	15.08
(ED_50_ + ED_50_)/8	7.48	0.05	7.54

## Data Availability

The data are contained within the article.
